# The Emerging Role of FUNDC1-Mediated Mitophagy in Cardiovascular Diseases

**DOI:** 10.3389/fphys.2021.807654

**Published:** 2021-12-17

**Authors:** Lei Liu, Yimei Li, Quan Chen

**Affiliations:** ^1^State Key Laboratory of Membrane Biology, Institute of Zoology, Chinese Academy of Sciences, Beijing, China; ^2^College of Life Sciences, University of Chinese Academy of Sciences, Beijing, China; ^3^Beijing Institute for Stem Cell and Regenerative Medicine, Beijing, China; ^4^Interdisciplinary Center of Cell Response, State key Laboratory of Medicinal Chemical Biology, College of Life Sciences, Nankai University, Tianjin, China

**Keywords:** mitochondria, mitochondria quality/dynamics, mitophagy, FUNDC1, cardiovascular diseases

## Abstract

Mitochondria are highly dynamic organelles and play essential role in ATP synthase, ROS production, innate immunity, and apoptosis. Mitochondria quality control is critical for maintaining the cellular function in response to cellular stress, growth, and differentiation Signals. Damaged or unwanted mitochondria are selectively removed by mitophagy, which is a crucial determinant of cell viability. Mitochondria-associated Endoplasmic Reticulum Membranes (MAMs) are the cellular structures that connect the ER and mitochondria and are involved in calcium signaling, lipid transfer, mitochondrial dynamic, and mitophagy. Abnormal mitochondrial quality induced by mitophagy impairment and MAMs dysfunction is associated with many diseases, including cardiovascular diseases (CVDs), metabolic syndrome, and neurodegenerative diseases. As a mitophagy receptor, FUNDC1 plays pivotal role in mitochondrial quality control through regulation of mitophagy and MAMs and is closely related to the occurrence of several types of CVDs. This review covers the regulation mechanism of FUNDC1-mediated mitophagy and MAMs formation, with a particular focus on its role in CVDs.

## Introduction

Mitochondria are highly dynamic, double membrane-bound organelles found in eukaryotic cells. They serve as the “powerhouses of the cell” that produce the ATP essential for all the cell’s activity (Friedman and Nunnari, 2014). In addition to ATP production, mitochondria are also involved in many essential biological processes, such as fatty acid β-oxidation, heme synthesis and iron–sulfur cluster biogenesis, innate immunity, calcium buffering, and programmed cell death (McBride et al., 2006). The efficient functioning of mitochondria is vital for their various roles in the cell, and when mitochondria were damaged, there were increased levels of reactive oxygen species (ROS), a by-product of energy generation (Huang and Manton, 2004; Zorov et al., 2006). Excessive ROS can disturb protein folding and cause mitochondrial DNA damage. In fact, mitochondria themselves are particularly vulnerable to the oxidizing damage of ROS to proteins and DNA due to inefficiency of the mtDNA repair system and restricted antioxidant capacity inside mitochondria (Zorov et al., 2014). Accumulation of dysfunctional mitochondria could generate retrograde stress signals to activate cellular programs including mitochondrial unfolding protein response (mtUPR) or leads to cell death through the release of proapoptotic proteins to the cytosol when the damage is beyond repair. Therefore, mitochondrial health is fundamental to cellular well-being (Wang, 2001). During evolution, cells have evolved elaborate mechanisms of mitochondrial quality control, including mitophagy, mitochondrial biogenesis, mitochondrial fission and fusion, and mtUPR (Fischer et al., 2012; Ni et al., 2015). The removal of mitochondria is balanced through the regulated mitochondrial biogenesis, which consists of biogenesis of new mitochondria and addition of new proteins/lipids to the mitochondrial (Attardi and Schatz, 1988).

Mitophagy is a selective form of autophagy that specific encloses damaged or unwanted mitochondria for autolysosomal degradation (Ding and Yin, 2012). Timely removal of damaged mitochondria is crucial for cell metabolism and functions and several pathways have been evolved to ensure that mitophagy can occur properly in response to a variety of cellular stimuli (Palikaras et al., 2018). Mitophagy regulatory pathways are classified as PINK1/Parkin-mediated ubiquitin-dependent pathway and the mitophagy receptor-dependent pathway (Palikaras et al., 2018). The ubiquitin-dependent mitophagy is regulated by the E3 ubiquitin ligase Parkin and mitochondria-located serine/threonine kinase PINK1 (Youle and Narendra, 2011). Under normal mitochondrial conditions, PINK1 is transported into mitochondria, where it is cleaved by mitochondrial proteases MPP and PARL and degraded by ubiquitin-proteasome system (Greene et al., 2012). When mitochondrial membrane potential is deficient, the protein import machinery of mitochondrial is blocked, leading to accumulation of PINK1 on the mitochondrial outer membrane (Youle and Narendra, 2011). PINK1 is autophosphorylated following mitochondrial membrane potential dissipation, which is essential for efficient mitochondrial localization of Parkin (Okatsu et al., 2012). Through its kinase activity, PINK1 phosphorylates Parkin at Ser65, and, as a result, Parkin becomes active to ubiquitinate multiple mitochondrial surface proteins, some of which serve as a signal for translocation of autophagy receptor OPTN to mitochondria and initializing mitophagy in a DFCP1 dependent manner (Wong and Holzbaur, 2014; Palikaras et al., 2018). Meanwhile, the ubiquitin and poly-ubiquitin chains on impaired mitochondria also can be phosphorylated by PINK1, which promotes association of Parkin with them and facilitates the activation of Parkin by PINK1 (Ordureau et al., 2014; Wauer et al., 2015). When Parkin is activated, more poly-ubiquitin chains are generated as substrates for PINK1 and the mitophagy signals are amplified (Ordureau et al., 2014; Wauer et al., 2015). Mitophagy receptors are typically mitochondrial proteins that contain an LC3 interaction region (LIR) motif that interacts with a critical autophagy protein LC3, leading to mitophagosomes expansion and engulfment the damaged mitochondria for removal (Liu et al., 2014). Thus, the interaction between the mitophagy receptors and LC3 is considered as a critical step in selecting mitochondria as the cargo (Palikaras et al., 2018). Several functional mitochondrial receptors have been identified, such as BNIP3, NIX, FUNDC1, PHB2, and BCL2-L-13 directly act in mitophagy (Palikaras et al., 2018). We have identified FUNDC1 as a mitophagy receptor to mediate hypoxia-induced mitophagy (Liu et al., 2012). We also found that FUNDC1 interacts with DRP1 to regulate mitochondrial fission which occurs at positions where ER tubules contacted mitochondria (Chen et al., 2016). Indeed, FUNDC1 has been reported to accumulate at ER-mitochondria contact sites by interacting with the ER membrane protein calnexin (Wu et al., 2016). MAMs have now been identified in species ranging from yeast to mammals. In mammalian cells, MAMs are required for a series of key cellular events including calcium transport and signaling, regulation of lipid synthesis and transport, the formation of autophagosomes, the regulation of mitochondrial dynamics, apoptosis, and inflammation (Rizzuto et al., 2012; Vance, 2014; Missiroli et al., 2018). Considering the apparent involvement of MAMs in multiple essential cellular processes, impaired contact between MAMs and mitochondria has been implicated in the pathology of several human diseases, including cardiovascular diseases (CVDs), neurodegenerative diseases, and metabolic diseases (Raturi and Simmen, 2013; Liu and Zhu, 2017; Theurey and Rieusset, 2017).

Maintaining mitochondrial quality is crucial in cardiomyocytes, as the mitochondria of the heart produce 6 kg/day of ATP, which account for roughly 8% of ATP consumption of the whole body, to continuously support the contraction–relaxation cycle within the myocardium (Brown et al., 2017; Sciarretta et al., 2018). The heart possesses the highest number of mitochondria of any tissue, mitochondria occupy almost 25–30% of the cardiomyocyte volume (Brown et al., 2017). Heart is particularly sensitive to oxidative stress. Damaged mitochondria produce less amount of ATP and more ROS, leading to establishment of a feed-forward loop whereby ROS-mediated oxidative damage to mitochondria favors more ROS generation and ultimately cardiomyocyte death (Szeto, 2006). Here, we will review current progress regarding the role of FUNDC1 in mitophagy and MAMs formation and the occurrence of some CVDs, including heart failure, ischemia–reperfusion, metabolic cardiomyopathy, and sepsis-induced cardiomyopathy.

## Fundc1 and Mitophagy

As an outer mitochondrial membrane protein, FUNDC1 contains a typical LIR domain in its N-terminal, which is exposed to the cytosol (Liu et al., 2012). The mitophagy induced by hypoxia was significantly inhibited when FUNDC1 was deficient, indicating that FUNDC1 functions as a mitophagy receptor to mediate damaged mitochondrial clearance during hypoxia exposure (Liu et al., 2012). The activity of FUNDC1 is stimulated by phosphorylation on Ser17 by ULK1 and dephosphorylation on Ser13 by PGAM5 upon hypoxia condition (Liu et al., 2012; Chen et al., 2014; Wu et al., 2014a). On the other hand, the function of FUNDC1 is compromised by phosphorylation on Tyr18 and Ser13 by Src and CK2, respectively, under normal conditions (Liu et al., 2012; Chen et al., 2014). In response to mitophagy stresses, the interaction between the mitochondrial phosphatase PGAM5 and FUNDC1 is enhanced, leading to dephosphorylation of FUNDC1 at Ser13, which enhances its interaction with LC3 to promote mitophagy (Chen et al., 2014). At normoxia, an antiapoptotic molecule, Bcl-xL binds to PGAM5 and inhibits the activity of PGAM5, whereas the protein level of Bcl-xL is reduced during hypoxic condition and more PGAM5 is released to bind to FUNDC1, leading to dephosphorylation of FUNDC1 and enhancement of mitophagy (Wu et al., 2014b). The activity of Bcl-xL is also regulated by PGAM5 through dephosphorylation at Ser62 of Bcl-xL, resulting in Bcl-xL activation and inhibition of apoptosis and FUNDC1-dependent mitophagy (Ma et al., 2020). Conversely, under some oxidative stress conditions, PGAM5 forms multimers and dissociates from Bcl-xL and dephosphorylates FUNDC1 to induce mitophagy (Ma et al., 2020). PGAM5 also can be cleaved by PARL, a rhomboid protease, and the cleaved form of PGAM5 boosts mitophagy through dephosphorylating of FUNDC1 (Sugo et al., 2018). However, the phosphorylation level of Tyr18 is more critical for the interaction between FUNDC1 and LC3 and the phosphatase of Tyr18 need to be identified (Kuang et al., 2016).

**Figure 1:**
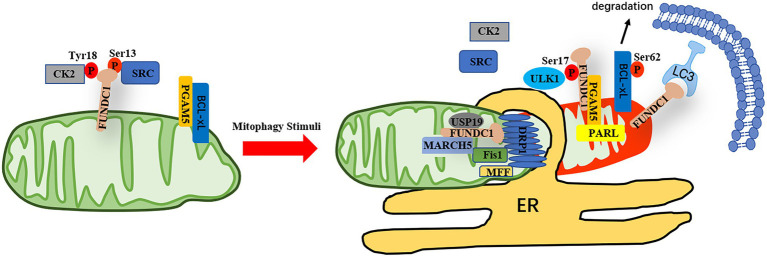
The regulation of FUNDC1-mediated mitophagy. Under normoxic conditions, the activity of FUNDC1 is inhibited due to phosphorylation of FUNDC1 at Ser13 and Tyr18 by CK2 or Src, respectively. The phosphatase PGAM5 dephosphorylates FUNDC1 at Ser13, which is inhibited by Bcl-xL in normal conditions. In response to mitophagy stimuli, Bcl-xL is degraded and PGAM5 is released to dephosphorylate FUNDC1 at Ser13 and the interaction of Src and CK2 with FUNDC1 is reduced, leading to dephosphorylation of FUNDC1 at Tyr18 and Ser13 and phosphorylation of BCL-xL at Ser62. PGAM5 also can be cleaved by PRAL and the interaction between cleaved form of PGAM5 and FUNDC1 is enhanced. Meanwhile, the kinase ULK1 is translocated to mitochondrial to phosphorylate FUNDC1 at Ser17. The protein level of FUNDC1 is regulated by MARCH5, whereas the interaction between FUNDC1 and DRP1 at MAMs is enhanced when FUNDC1 is deubiquitinated by USP19. Altogether, the function of FUNDC1 is regulated by reversible phosphorylation and ubiquitination, favoring segregation of damaged mitochondria and mitophagy.

FUNDC1 can interact with mitochondrial fusion protein OPA1 and mitochondrial fission protein Drp1 and these interactions are of functional importance to the mediate hypoxia-induced mitophagy (Chen et al., 2016). When mitophagy is induced, FUNDC1 dissociates from OPA1 and associates with Drp1, which subsequently leads to mitochondrial fission and mitophagy (Chen et al., 2016). The protein level of FUNDC1 is also controlled by a ubiquitin-protein ligase, MARCH5, which is essential for the desensitization of mitochondria to mitophagy during early stage of hypoxia (Chen et al., 2017a,b). On the other hand, the deubiquitinase USP19 binds to deubiquitinates FUNDC1 at MAMs and stabilizes FUNDC1 at the ER-mitochondria contact sites, which causes recruitment of activated Drp1 to MAMs and mitochondrial fission (Chai et al., 2021; Figure 1).

## Fundc1 and Heart Failure

Heart failure is a complex disease in which the heart has difficulty pumping enough oxygenated blood to support surrounding tissues. Because of a growing and aging population, the total number of patients living with heart failure is increasing (Groenewegen et al., 2020). Heart failure is a costly and devastating disease and 1 in 5 people over 40 years of age will develop heart failure during their lifetime; therefore, the cost and public health burden are staggering (Pfeffer et al., 2019). The pathophysiology of heart failure still remains unclear; however, mitochondrial dysfunction has been implicated in the progression of heart failure (Zhou and Tian, 2018). Besides energy supplier failures, redox imbalance, ROS generation, and mitochondrial Ca^2+^ imbalance induced by mitochondrial dysfunction also contribute to hear failure (Sorescu and Griendling, 2002; Bayeva et al., 2013; Rosca and Hoppel, 2013; Zhou and Tian, 2018).

Wu et al. found that FUNDC1 is involved in maintaining cardiac function through regulation of MAMs and Ca2+ homeostasis (Wu et al., 2017). Moreover, they also demonstrated that disruption of this protein function leads to cardiac dysfunction and heart failure (Wu et al., 2017). They found that FUNDC1 is localized in MAMs through binding to IP3R2, a major Ca^2+^ release channels (CRCs) on the ER (Wu et al., 2017). FUNDC1 ablation results in MAMs disruption and IP3R2 protein level reduction, which lead to reduced levels of Ca^2+^ both in mitochondria and cytosol and compromised mitochondrial functions (Wu et al., 2017). Mechanistically, they found that reduction of intracellular Ca^2+^ levels induced by Fundc1 disruption inhibits the binding of CREB in the Fis1 promoter and reduces the expression of Fis1 and mitochondrial fission (Wu et al., 2017). They also showed that cardiomyocyte-specific *Fundc1* gene knockout instigates abnormal mitochondrial dynamic, mitochondrial function impairment, and heart failure (Wu et al., 2017). Indeed, the localization of FUNDC1 in MAMs was first observed in the study of the role of MAMs in mitochondrial fission and mitophagy mediated by FUNDC1 (Wu et al., 2016). Wu et al. reported that FUNDC1 accumulates at the MAM by interaction with an ER membrane protein calnexin and DRP1 is recruited to the MAMs to mediate mitochondrial fission by association with FUNDC1 when mitophagy is initiated by cellular stress (Wu et al., 2016). These studies indicated that FUNDC1 is a MAMs protein and plays important role in mitochondrial dynamic, mitophagy, and Ca2+ homeostasis through interaction with related ER proteins. Moreover, FUNDC1 also plays important role in pressure overload-induced left ventricular hypertrophy and dysfunction and alpha-lipoic acid (α-LA) reduces the degree of transverse aortic constriction (TAC)-induced heart failure in a FUNDC1-related manner (Li et al., 2020). ALDH2 activity and expression are restored by α-LA in TAC-induced heart failure model, which in turn increases the expression of FUNDC1 in a NRF1-dependent manner (Li et al., 2020). Their finding about the regulation mechanism of *FUNDC1* expression is consistent with our recent work. We also demonstrated that the expression of *FUNDC1* is regulated by PGC-1α/NRF1 cascade, which couples FUNDC1-dependent mitophagy with mitochondrial biogenesis and contributes to adaptive thermogenesis (Liu et al., 2021).

## Fundc1 and Ischemia–Reperfusion Injury

Coronary heart disease (CHD) is the most common type of CVDs and is manifest as an acute coronary syndrome (ACS). Although the mortality of CHD has gradually reduced over the past decades in western countries due to the in-time reperfusion medical strategies, it still causes about one-third of all deaths in people older than 35 years (Sanchis-Gomar et al., 2016; Yang et al., 2019). However, the mortality from CHD is expected to continue increasing in developing countries and CHD is a major cause of death and disability in developed countries (Sanchis-Gomar et al., 2016). The high mortality is caused by myocardial injury that occurs when the ischemic heart is re-establishment of blood flow, called reperfusion (Silvis et al., 2020). Ischemia–reperfusion (I/R) injury is a significant problem accompanied by a burst of ROS generation and aggravation of myocardial injury due to cardiac reperfusion strategies following ACS (Ma et al., 2012). Dysfunctional mitochondria activate mitochondrial remodeling and cellular responses that modulate the balance between cell death and recovery and have been proposed to play a crucial role in mediating cardiac I/R injury (Lesnefsky et al., 2017). Given the pivotal role for mitophagy in mitochondrial quality control and recent reports supporting a connection between mitophagy and cardiac I/R injury. However, the relationship between mitophagy and cell fate in I/R injury is complicated, which is dependent on the types and levels of mitophagy and the phase of I/R process. In general, cardiac injury is increased during I/R when mitophagy is inhibited, indicating mitophagy as a protective process that decreases cardiac injury. In contrast, in some cases, excessive mitochondrial elimination in response to I/R can increase cardiomyocyte death (Lesnefsky et al., 2017).

FUNDC1-depedent mitophagy plays an important role in cardiac I/R injury through regulation of platelet activation (Zhang et al., 2016; Zhou et al., 2017a). The mitophagy and platelet activation induced by cardiac I/R are inhibited in platelet specific Fundc1 knockout mice, leading to protection from heart injury (Zhang et al., 2016). Melatonin also can suppress platelet hyperactivity and cardiac I/R injury *via* inhibition of FUNDC1-mediated mitophagy in a PPARγ-dependent manner (Zhou et al., 2017a). As a kinase, CK2α can phosphorylate FUNDC1 at Ser13 and inhibit the mitophagy induced by FUNDC1 (Chen et al., 2014; Wu et al., 2014b). The expression of CK2α and the phosphorylation of FUNDC1 is upregulated following cardiac I/R injury, this suggests that the activity of FUNDC1 is involved in cardiac I/R injury (Zhou et al., 2018b). FUNDC1-mediated mitophagy is reversed in CK2α knockout mice during I/R process, which can reverse mitochondrial membrane potential, reduce ROS production, and protect against cardiac reperfusion injury (Zhou et al., 2018b). The upregulation of CK2α in response to I/R is controlled by NR4A1, a nuclear receptor subfamily protein (Zhou et al., 2018a). NR4A1 is significantly upregulated following cardiac I/R injury, leading to activation of CK2α and Mff and inhibition of FUNDC1 (Zhou et al., 2018a). Ultimately, excessive fission and less mitophagy lead to impaired mitochondrial quality and function, contributing to mitochondrial apoptosis during cardiac I/R (Zhou et al., 2018a). Similarly, FUNDC1-mediated mitophagy also involves in ischemia preconditioning (IPC)-mediated renoprotection and IPC-offered renoprotection is diminished when *Fundc1* is specific knockout in proximal tubule (Wang et al., 2020). Ripk3 plays essential role in necroptosis and loss of Ripk3 protects the mitochondria against cardiac I/R injury (Zhou et al., 2017b). Ripk3 interacts with FUNDC1 and FUNDC1-mediated mitophagy also closely involves in Rpik3-related necroptosis and apoptosis (Zhou et al., 2017b). FUNDC1-dependent mitophagy is activated during ischemia to eliminate damaged mitochondria and protect cardiomyocytes apoptosis (Zhou et al., 2017b), whereas the expression of Ripk3 is elevated during reperfusion, which inhibits FUNDC1-mediated mitophagy and increases cell death in isolated cardiomyocytes (Zhou et al., 2017b). The role of FUNDC1 in myocardial protection was also investigated in cells models, Jiang et al. found that irisin inhibits mitochondrial dysfunction in LPS-treated H9C2 cells through enhancing FUNDC1-dependent mitophagy (Jiang et al., 2021).

## Fundc1 and Metabolic Cardiomyopathy

Excessive fat accumulation, which has negative health consequences, is associated with metabolic complications of obesity that often referred to as the metabolic syndrome, including insulin resistance and type 2 diabetes, hypertension, atherosclerosis, and premature heart disease (Singla et al., 2010; Nishida and Otsu, 2017). Metabolic cardiomyopathy develops in numerous pathological conditions and is tied with systemic metabolic disorders and is acquired during adulthood or congenital (Nishida and Otsu, 2017). Metabolic cardiomyopathy, particularly, is characterized by the presence of functional and structural changes in the heart muscle and intermediary fibrosis without coronary artery disease or hypertension (Nishida and Otsu, 2017). Metabolic cardiomyopathy is a major cause of heart failure and death in patients with metabolic syndrome (Ashrafian et al., 2007; Lavie et al., 2019). There is compelling evidence to suggest that mitochondrial dysfunction is a key contributor to the occurrence of metabolic cardiomyopathy, resulting in unfavorable myocardial structural and functional alterations (Guertl et al., 2000; Ren et al., 2010; Galloway and Yoon, 2013; Meyers et al., 2013; Sverdlov et al., 2016; Liu et al., 2017).

It is reported that FUNDC1-mediated MAM formation plays essential role in diabetes mellitus-related cardiomyopathy (DMCMP; Wu et al., 2019). The expression of FUNDC1 is significantly elevated in cardiac tissues from diabetic donors in comparison with nondiabetic donors, implicating that the level of FUNDC1 is correlated with the occurrence of DMCMP (Wu et al., 2019). Furthermore, the expressions of FUNDC1 and IP3R2 and the levels of MAMs are elevated in high glucose (HG)-treated mouse neonatal cardiomyocytes (Wu et al., 2019). Elevations of MAMs formation and endoplasmic reticulum mitochondrial Ca2+ flux and impairment of mitochondrial function in response to HG treatment are recovered or accentuated when Fundc1 or IP3R2 is knockdown or overexpression in mouse neonatal cardiomyocytes (Wu et al., 2019). They also found the interaction between FUNDC1 and IP3R2 is significantly increased in streptozotocin-induced diabetic mice heart, leading to stabilization of IP3R2 and mitochondrial Ca2+ increase and mitochondrial dysfunction (Wu et al., 2019), whereas diabetes mellitus-induced MAMs formation and mitochondrial Ca^2+^ elevation and mitochondrial fission, and apoptosis are restored when Fundc1 is specifically knockout in mouse heart (Wu et al., 2019). Overall, their findings indicated that FUNDC1-related MAMs formation and mitochondrial Ca^2+^ elevation play critical role in DMCMP and FUNDC1 is a novel and valid target for DMCMP treatment. However, controversial finding was noted for the role of FUNDC1 under obesity-induced cardiomyopathy. Ren et al. reported that FUNDC1-mediated mitophagy plays a protective role in the development of cardiomyopathy induced by obesity (Ren et al., 2020). They revealed that high-fat diet-induced cardiac remodeling, mitochondrial dysfunction and Ca^2+^ overload, cell death, and IP3R3 increase are aggravated when FUNDC1 is absent in mice (Ren et al., 2020). Mechanistically, they found that FUNDC1 interacts with an ubiquitin ligase complex FBXL2, which is a E3 ligase of IP3R3. When FUNDC1 is deficient, the degradation of FBXL2 is accelerated, leading to stabilization of IP3R3 and mitochondrial Ca^2+^ overload and mitochondrial dysfunction.

## Fundc1 and Sepsis-Induced Cardiomyopathy

Cardiac dysfunction is a well-recognized manifestation of organ dysfunction in severe sepsis and septic shock, acting as a significant contributor to morbidity and mortality (Flynn et al., 2010). Cardiac dysfunction in sepsis is characterized by left ventricular dilatation, reduced ejection fraction, and contractility (Flynn et al., 2010). In most of the cases, sepsis-induced cardiomyopathy (SICM) is a reversible myocardial dysfunction that typically recovers in 7–10 days (Sato and Nasu, 2015). However, many uncertainties exit regarding the pathophysiological mechanisms of SICM, a growing body of evidence suggests that mitochondrial dysfunction, bioenergetic failure, and metabolic derangements play pivotal roles in pathogenesis of this condition, which place the cardiomyocytes at risk of energy depletion (Rudiger and Singer, 2007; Stanzani et al., 2019). Cytopathic hypoxia refers to the phenomenon of decrease in the rate of oxygen consumption by septic muscle despite adequate oxygen delivery, leading to oxidative phosphorylation uncoupling and diminished ATP content in cardiomyocytes (Flynn et al., 2010). Decreased activity and expression of mitochondrial respiratory chain enzymes and structural abnormalities in the mitochondria are highly correlated with deterioration of cardiac function during sepsis (Chen et al., 2003).

Wang et al.’s work showed that activation of FUNDC1-mediated mitophagy by UA, a mitophagy inducer, attenuated SICM in LPS-treated mice model (Wang et al., 2021). They found that the elevation of the levels of cardiac injury markers by LPS treatment is inhibited by UA administration in control mice but not in cardiomyocyte-specific FUNDC1 knockout mice (Wang et al., 2021). The expression of mtUPR-related genes is significantly upregulated when FUNDC1 is deficient in LPS-treated heart, indicating that mtUPR is activated when mitophagy is blocked in response to LPS treatment (Wang et al., 2021). Activation of mtUPR by oligomycin administration alleviated mitochondrial and myocardial dysfunction and these beneficial effects are diminished when FUNDC1 is deleted in mice cardiomyocyte (Wang et al., 2021). Although mtUPR activation has no effect on mitophagy, mitophagy-mediated protection of mitochondria and cardiomyocytes is partly weakened when mtUPR is inhibited by knockdown the expression of ATF6 in human ventricular cardiomyocyte cell line (Wang et al., 2021). Similarly, Jiang et al. also found that irisin treatment also alleviates septic cardiomyopathy in H9C2 cells induced by LPS in a FUNDC1-dependent manner (Jiang et al., 2021). In summary, their works demonstrated that coordination of FUNDC1-mediated mitophagy and mtUPR plays a key role in protection against LPS-induced cardiomyopathy and shed light on a new avenue toward the understanding and treatment of SICM. Our previous work has revealed that FUNDC1-mediated mitophagy suppressed hepatocarcinogenesis by suppression of inflammasome activation by diethylnitrosamine (DEN) in liver (Li et al., 2019). Interestingly, two recently reports have also demonstrated that FUNDC1-mediated mitophagy suppresses LPS plus nigericin-mediated IL-1β production or apoptosis in the lung induced by LPS through inhibition of ROS-NLRP3, indicating that both FUNDC1-mediated mitophagy and its related inflammatory response are involved in SICM development (Huang et al., 2020; Pan et al., 2021).

## Conclusion

The relevance of FUNDC1 in many CVDs is emerging. Mitochondrial damage and dysfunction are prominent pathological mechanisms of CVDs. Mitochondrial dysfunction can derive from defects on mitochondrial biogenesis, mitophagy, dynamics and abnormal MAMs, leading to impaired respiratory chain function and reduced ATP production, increased sensitivity to the apoptotic stimuli, and mitochondrial calcium imbalance. Abnormal function of mitochondria often leads to abnormal function of cardiomyocytes and CVDs. As a mitophagy receptor, FUNDC1 also plays critical role in MAMs formation and mitochondrial Ca^2+^ homeostasis; therefore, there is no doubt that FUNDC1 is closely related to the pathogenesis of many CVDs. In most CVDs, FUNDC1-mediated mitophagy plays a protective role and many CVDs are aggravated when FUNDC1 is deficient. However, the role of FUNDC1-related MAMs in CVDs is controversial. For example, FUNDC1 deficiency caused heart failure in mice due to reduced MAMs formation and mitochondrial Ca^2+^ level (Wu et al., 2017); conversely, the heart function is restored in STZ-induced diabetes mellitus mice when FUNDC1 is deleted, owing to decreased MAMs formation and mitochondrial Ca^2+^ level (Wu et al., 2019). Whereas in obesity-induced cardiopathy, mitochondrial dysfunction and Ca^2+^ overload are worsened when FUNDC1 is absent in mice (Ren et al., 2020). These significant discrepancies perhaps reflect variability in the experimental models employed and the complicated role of FUNDC1 in mitophagy control and MAMs formation.

The induction of FUNDC1-mediated mitophagy can potently clean damaged mitochondria and maintain normal mitochondria function for cell homeostasis, which makes FUNDC1 as a promising therapeutic target for CVDs. Screening the small molecular compounds targeting to FUNDC1-mediated mitophagy, which would restore normal mitophagy fluxes, will provide new opportunities for CVDs prevention and treatment. FUNDC1-mediated mitophagy also plays a critical role in mitochondrial network formation during adult cardiac progenitor cells differentiation (Lampert et al., 2019). FUNDC1-mediated mitophagy enhancement will also contribute to the success of cell-based therapies in CVDs. Further studies are required to investigate whether the FUNDC1-mediated mitophagy and MAMs formation have mutual effects on the progression of many CVDs, which is also essential for these CVDs treatment.

## Author Contributions

LL wrote the original manuscript. YL and QC contributed to the manuscript revision. All authors approved the submission.

## Funding

This work was supported by Grants 2020YFA0803702 and 2019YFA0508601 from the Ministry of Science and Technology of China and the National Natural Science Foundation of China (91849201, 31790404, 91854105, and 31970716).

## Conflict of Interest

The authors declare that the research was conducted in the absence of any commercial or financial relationships that could be construed as a potential conflict of interest.

The reviewer YZ declared a shared affiliation with one of the authors QC, to the handling editor at time of review.

## Publisher’s Note

All claims expressed in this article are solely those of the authors and do not necessarily represent those of their affiliated organizations, or those of the publisher, the editors and the reviewers. Any product that may be evaluated in this article, or claim that may be made by its manufacturer, is not guaranteed or endorsed by the publisher.
